# Molecular and Pathophysiological Mechanisms Leading to Ischemic Heart Disease in Patients with Diabetes Mellitus

**DOI:** 10.3390/ijms26093924

**Published:** 2025-04-22

**Authors:** Stefan Juricic, Jovana Klac, Sinisa Stojkovic, Milorad Tesic, Ivana Jovanovic, Srdjan Aleksandric, Milan Dobric, Stefan Zivkovic, Bojan Maricic, Dejan Simeunovic, Ratko Lasica, Miodrag Dikic, Marko Banovic, Branko Beleslin

**Affiliations:** 1 Clinic for Cardiology, University Clinical Center of Serbia, 11000 Belgrade, Serbia; sstojkovi@mts.rs (S.S.); misa.tesic@gmail.com (M.T.); ivana170679@gmail.com (I.J.); srdjanaleksandric@gmail.com (S.A.); drdejansimeunovic@gmail.com (D.S.); miodrag.dikic@gmail.com (M.D.); markobanovic71@gmail.com (M.B.); 2 Department of Cardiology, Emergency Center, University Clinical Center of Serbia, 11000 Belgrade, Serbia; jovanablagojevic718z@gmail.com (J.K.); drlasica@gmail.com (R.L.); 3 School of Medicine, University of Belgrade, 11000 Belgrade, Serbia; iatros007@gmail.com; 4 Dedinje Cardiovascular Institute, 11000 Belgrade, Serbia; zivkovic.stefan@gmail.com; 5 Clinic of Cardiology, University Clinical Center Nis, 18000 Nis, Serbia; bokimaricic@gmail.com

**Keywords:** atherosclerosis, diabetes mellitus, hyperglycemia, oxidative stress, dyslipidemia, chronic inflammation

## Abstract

Coronary atherosclerosis in patients with diabetes mellitus is the most significant pathophysiological mechanism responsible for ischemic heart disease. Atherosclerosis in diabetes is premature, more diffuse, and more progressive, and it affects more coronary blood vessels compared to non-diabetics. Atherosclerosis begins with endothelial dysfunction, continues with the formation of fatty streaks in the intima of coronary arteries, and ends with the appearance of an atherosclerotic plaque that expands centrifugally and remodels the coronary artery. If the atherosclerotic plaque is injured, a thrombus forms at the site of the damage, which can lead to vessel occlusion and potentially fatal consequences. Diabetes mellitus and atherosclerosis are connected through several pathological pathways. Among the most significant factors that lead to atherosclerosis in diabetics are hyperglycemia, insulin resistance, oxidative stress, dyslipidemia, and chronic inflammation. Chronic inflammation is currently considered one of the most important factors in the development of atherosclerosis. However, to date, no adequate anti-inflammatory therapeutic measures have been found to prevent the progression of the atherosclerotic process, and they remain a subject of ongoing research. In this review, we summarize the most significant pathophysiological mechanisms that link atherosclerosis and diabetes mellitus.

## 1. Introduction

Atherosclerosis is a diffuse chronic inflammatory disease of the arterial blood vessels that primarily affects large and medium-sized arteries [1]. The process of atherosclerosis begins with endothelial damage to the blood vessel, most commonly due to turbulent blood flow, hypertension, and oxidative stress, and continues with lipid accumulation and chronic inflammation [2,3,4].

**Figure 1:**
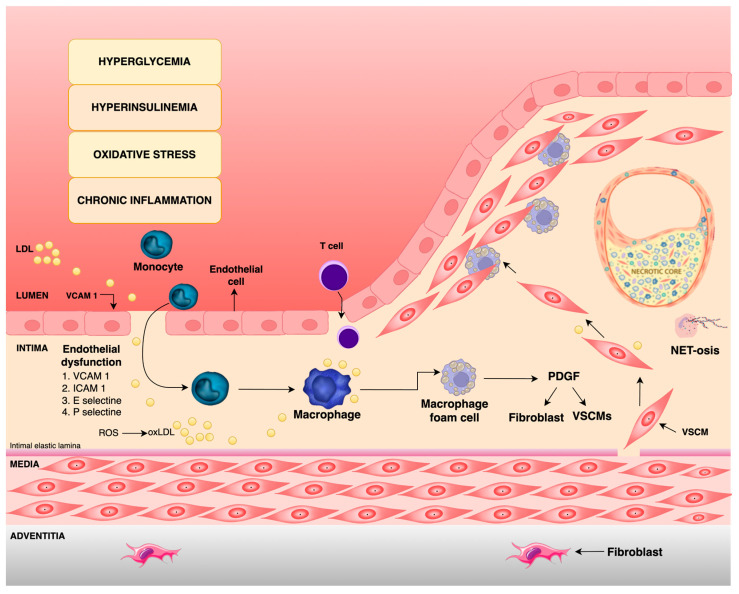
Schematic representation of the pathophysiological connection between diabetes mellitus and atherosclerosis. Hyperglycemia, dyslipidemia, oxidative stress, and chronic inflammation in patients with diabetes cause a spectrum of physiological changes in the endothelium of blood vessels, primarily leading to its damage. A damaged endothelium in the presence of pro-inflammatory cytokines increases leukocyte adhesion through the help of adhesion molecules (ICAM-1, VCAM-1, E-selectin, and P-selectin). Leukocytes, predominantly monocytes and T lymphocytes, more easily adhere and pass through the intimal layer of the blood vessel, where they differentiate into macrophages. Macrophages phagocytize LDL, becoming foam cells. Foam cells secrete growth factors (such as PDGF), which stimulate the proliferation of fibroblasts and smooth muscle cells. The figure also shows how neutrophils in a hyperglycemic state activate the process of NETosis, which helps in pathogen elimination but also contributes to further tissue damage. VCAM 1, Vascular Cell Adhesion Molecule; ICAM 1, Intercellular Adhesion Molecule; PDGF, Platelet-Derived Growth Factor; VSCMs, Vascular Smooth Muscle Cells; LDL, Low-Density Lipoprotein; oxLDL, oxidized LDL; ROS, Reactive Oxygen Species.

Endothelial damage in the presence of pro-inflammatory agents, such as interleukin 1 (IL-1), Tumor Necrosis Factor (TNF), endotoxins, and advanced glycation end-products (AGEs) enhances the expression of adhesion molecules: vascular cell adhesion molecule (VCAM-1), intercellular adhesion molecule (ICAM-1), platelet endothelial cell adhesion molecule (PECAM-1), E-selectin, and P-selectin for leukocyte adhesion [1,5,6]. Leukocytes from the blood, predominantly monocytes, more easily adhere to and penetrate the intimal layer of the blood vessel wall and they transform into macrophages. Macrophages then phagocytize low-density lipoproteins (LDLs) from the blood, and in this way, they become foam cells, which form the basis for the development of early atherosclerotic lesions—“fatty streak” [1,7,8]. Endothelial cells and macrophages oxidatively modify LDL in such a way that normal LDL receptors no longer recognize them, and they are mostly taken up through non-specific phagocytosis [8,9]. Oxidized LDL (oxLDL) further stimulates endothelial cells to produce monocyte chemoattractant factor, which will lead to the recruitment of other leukocytes (T lymphocytes and dendritic cells) into the intima of the blood vessel [8,10]. According to the latest findings, vascular smooth muscle cells (VSMCs) in addition to macrophages, can also take up oxLDL and thus become foam cells [1,8,11,12]. One of the roles of foam cells is to secrete numerous proteolytic enzymes, as well as release growth factors such as platelet-derived growth factor (PDGF), which stimulates the proliferation of fibroblasts and smooth muscle cells [1,13]. VSMCs, stimulated by foam cells, are pulled from the media to the intima through chemotaxis, where further division, synthesis, and secretion of the connective tissue matrix occur, leading to the formation of the atherosclerotic plaque [1,14] (Figure 1). Phenotypic changes of VSMCs, specifically the transition from a contractile to a synthetic phenotype, are key to the formation and progression of atherosclerotic plaques. This process, which involves increased proliferation, migration, and adhesion of VSMCs, contributes to the development of atherosclerosis and is particularly pronounced in diabetic patients, increasing the risk of atherosclerotic events and restenosis. Several studies have shown that proinflammatory cytokines (such as IL-1β, which is chronically activated in T2DM) can also induce a change in VSMC to a synthetic phenotype [15,16]. Another factor to consider regarding the behavior of VSMCs in DM is the disruption of insulin signaling. Physiologically, insulin maintains VSMCs in a quiescent state through the activation of the phosphoinositide 3-kinase pathway (PI3K), while simultaneously promoting their migration through the activation of the Mitogen-Activated Protein Kinase pathway (MAPK). In conditions of insulin resistance, impaired PI3K signaling and intact MAPK signaling disrupt normal function, resulting in the pathological stimulation of VSMC migration and contributing to the progression of the atherosclerotic plaque [15].

The atherosclerotic plaque is a structure composed of lipids, proteins, and accumulated cells, and it is covered by a fibrous cap that serves as a barrier between the plaque and the lumen of the arterial blood vessel. Such a plaque can narrow the lumen of the blood vessel and lead to tissue ischemia [1,17]. Additionally, the plaque can become prone to rupture or fissuring under the action of matrix metalloproteinases (MMPs), which degrade the extracellular matrix, thereby leading to the exposure of the subendothelial space to the bloodstream. At the site of the lesion, platelet migration and activation of coagulation factors, predominantly factor VII, occur, which, together with fibrin, form a thrombus that can cause vessel occlusion [1,4,18,19,20].

### Methodology of the Review

The main goal of this review was to analyze the pathophysiological mechanisms linking the harmful effects of hyperglycemia with atherosclerosis in diabetic patients. The search for this review was conducted in the following databases: PubMed and Google Scholar. The following keywords were used during the search: hyperglycemia, insulin resistance, oxidative stress, dyslipidemia, chronic inflammation, NETosis, and miRNA. The search was limited to studies published between 1993 and 2025. During the writing of this review, we used clinical, preclinical, randomized controlled, and cohort studies. Studies that examined the relationship between insulin resistance and atherosclerosis, as well as studies that explored the impact of glycemic control on the development of complications in type 1 diabetes mellitus, were included. Only studies written in English were used, with the majority of data being gathered from papers published in the last 10 years. Abstracts or non-peer-reviewed papers were not used.

## 2. The Connection Between Atherosclerosis and Diabetes Mellitus

Diabetes mellitus and cardiovascular diseases often occur together. Cardiovascular diseases are very common in patients with diabetes, regardless of the type of diabetes, and it is estimated that morbidity and mortality are 2–4 times higher than in non-diabetics [21,22]. Such high morbidity and mortality from cardiovascular diseases is not only the result of diabetes itself, but also a result of the synergistic effect of other risk factors. The main metabolic, atherogenic, and prothrombotic factors, which lead to atherosclerosis in diabetics, are hyperglycemia, insulin resistance, oxidative stress, dyslipidemia, and chronic inflammation [4,21].

## 3. Hyperglycemia in Diabetic Patients

The toxic effects of hyperglycemia are complex; some manifest immediately, while others are the result of prolonged exposure to high blood sugar levels.

### 3.1. Acute Effect of Hyperglycemia

The acute effect of hyperglycemia leads to direct endothelial cell damage, creating conditions for vasoconstriction and the expression of vasoactive molecules, and facilitates activation of coagulation [23]. In the pathogenesis, altered activity of protein kinase C (PKC) occupies a special place. It is believed that increased glucose uptake by VSMCs leads to the increased synthesis of diacylglycerol, which contributes to the activation of PKC [21,24]. PKC has numerous pro-atherogenic effects, such as reduced production of nitric oxide (NO), a potent vasodilator, stimulation of endothelin production, a strong vasoconstrictor, leading to endothelial cell damage and increased permeability, and increased production of cytokines and extracellular matrix molecules [24,25]. There are several different isoforms of PKC that contribute at various levels to the development and progression of atherosclerosis [21]. For example, PKCα and PKCβ stimulate the activation of endothelial cells to express adhesion molecules ICAM-1 and VCAM-1, while PKCβ and PKCδ regulate the uptake of oxLDL through receptors on macrophages, thereby contributing to the formation of foam cells [21,26]. Additionally, PKCδ is the main regulator of VSMC proliferation and migration, which is a key step in plaque formation, while on the other hand, it also stimulates VSMC apoptosis, leading to plaque vulnerability [25]. PKC can also lead to the activation of nicotinamide adenine dinucleotide phosphate oxidase 2 (NOX2), which is one of the main sources of reactive oxygen species (ROS) [24]. These data suggest that different isoforms of PKC could be therapeutic targets to reduce the progression of atherosclerosis and its complications.

### 3.2. Prolonged Effect of Hyperglycemia

The prolonged effect of hyperglycemia is called metabolic memory [4]. This concept originated from the Diabetes Control and Complications Trial (DCCT) and Epidemiology of Diabetes Interventions and Complications (EDICs) studies and refers to the long-term consequences of poor early glycemic control in type 1 diabetes, even when better blood sugar regulation is achieved later [27]. One of the possible mechanisms of this effect is the formation of AGEs, which accumulate in patients with long-standing diabetes [4,28]. AGEs affect the activation of endothelial cells and the surface expression of adhesion molecules, thereby leading to the easier adhesion and entry of monocytes/macrophages into the subendothelial space during the early stages of plaque formation [1,4,9]. An important effect of AGEs is the oxidation of LDL particles, which is considered one of the atherogenic modifications of LDL [4,8]. When AGE binds to its RAGE receptor (receptor for advanced glycation end products), it leads to the release of transforming growth factor beta (TGF-β). TGF-β further leads to the migration and proliferation of VSMCs and the production of the extracellular matrix and ROS [4,29,30,31]. AGE/RAGE signaling also stimulates the increased expression of the lectin-like oxidized low-density lipoprotein receptor (LOX-1) in endothelial cells, leading to the enhanced uptake of oxLDL [32]. On the other hand, AGE increases the production of ROS and stimulates the activation of nuclear factor kappa B (NF-kB), which contributes to the production of inflammatory cytokines [24,33]. It has also been shown that AGEs enhance vasoconstriction by increasing the levels of endothelin-1 (ET-1), which leads to the inhibition of endothelial nitric oxide synthase (eNOS) and the formation of NO, a important vasodilator [4,21,31,34]. Research has shown that AGEs lead to neovascularization in plaques, thereby contributing to bleeding and plaque instability. Furthermore, AGEs promote thrombus formation by stimulating the synthesis of plasminogen activator inhibitor (PAI-1) [31,35]. Increased formation of AGEs leads to pericyte damage, endothelial dysfunction, and enhanced platelet aggregation, making them one of the most significant procoagulants in states of hyperglycemia [24,36].

Whether it is acute or chronic hyperglycemia, the most common mechanism that leads to cellular damage is the increased production of ROS in tissues. Initially, it was believed that ROS in hyperglycemic conditions were generated due to increased electron flow through the mitochondria. However, it is now considered that ROS production is independent of the mitochondria, with the main role played by the activation of NOX2 [24,37].

### 3.3. The Impact of Hyperglycemia on Platelet Function

During hyperglycemia, glucose uptake in platelets is increased since this uptake in these cells is not insulin-mediated. There are several harmful effects of hyperglycemia that can disrupt platelet function, thereby contributing to the progression of atherosclerosis and the destabilization of the atherosclerotic plaque [31].

1. In platelets, as well as in endothelial cells, elevated glucose levels lead to the activation of PKCβ, a reduction in NO production, and an increase in ROS synthesis, predominantly superoxide (O_2_^−^) [24,25,31].
2. Excessive ROS production in platelets disrupts intracellular calcium homeostasis, leading to an increase in calcium levels, which can cause changes in platelet shape, impair secretion and aggregation, and enhance thromboxane production [31,38].
3. In a hyperglycemic state, there is increased expression of glycoprotein Ib (GpIb) on the surface of platelets, which has an affinity for binding to von Willebrand factor (vWF) and GpIIb/IIIa, thereby promoting platelet interaction with fibrin [35].
4. Activated platelets release TGF-β, which stimulates the production of interstitial collagen, leading to the thickening of the fibrous cap [1,39].

### 3.4. The Impact of Hyperglycemia on the Coagulation Process

In addition to its effects on platelet function, diabetes also increases blood coagulability, which contributes to the formation of thrombotic artery occlusion [35]. Research has shown that in diabetics, there is an increased expression of angiotensin II (AT2), a potent vasoconstrictor, which increases the synthesis of PAI-1, a strong atherogenic and thrombogenic factor [31,35]. Additionally, diabetes increases the expression of tissue factor and vWF, strong procoagulants, as well as plasma coagulation factors such as factor VII, while decreasing the levels of endogenous anticoagulants, such as antithrombin III (AT III) and protein C (PC) [18,23,31,35]. At the same time, natural anticoagulants such as tissue plasminogen activator (t-PA) have lower plasma concentrations, while AGEs modify the sites of endothelial receptors for t-PA [35].

Therefore, in diabetes, there is an increased tendency for coagulation, and along with reduced fibrinolysis, it contributes to the formation and persistence of thrombus. It is still unclear whether fibrinolytic dysfunction in diabetes is primary or represents a response to vascular injury.

Studies have also indicated a significant role of hyperglycemia in the pathogenesis of coronary microvascular dysfunction and the development of restenosis in patients with ST-Elevation Myocardial Infarction (STEMI). Through its detrimental effects on endothelial function, induction of inflammation, and oxidative stress, hyperglycemia may contribute to the onset of Ischemia and Nonobstructive Coronary Arteries (INOCA) and an increased risk of rehospitalization due to anginal symptoms [40].

### 3.5. Hyperglycemia and the Polyol Pathway

**Figure 2:**
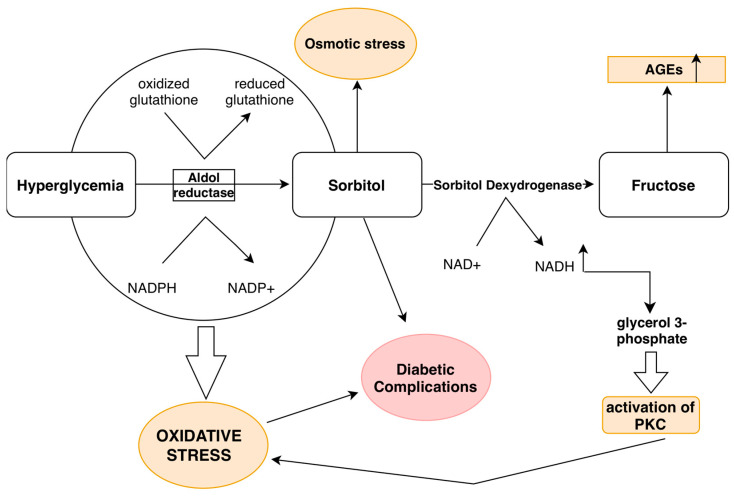
Schematic representation of the activation of the polyol pathway contributing to the development of oxidative stress and chronic inflammation. Final products of the activation are marked on the figure (marked in orange color), while all of them represent complications of diabetes (marked in red color). In conditions of hyperglycemia, excess glucose is converted to sorbitol with the help of the enzyme aldose reductase, which is then further converted to fructose with the help of the en-zyme sorbitol dehydrogenase. Fructose, as the end product of this metabolic pathway, contributes to the increased synthesis of (AGEs). In the polyol pathway, the conversion of glucose to sorbitol and fructose, along with the increase in NADH levels, leads to a reduction in NADPH levels, which lowers the production of glutathione, a key antioxidant, thereby increasing oxidative stress. Addi-tionally, the activity of sorbitol dehydrogenase leads to an increase in NADH levels, which, through glycerol-3-phosphate, results in the activation of protein kinase C (PKC). NADPH, nico-tinamide adenine dinucleotide phosphate; NADH, nicotinamide adenine dinucleotide; AGEs, ad-vanced glycation end-products; PKC, protein kinase C.

It should not be forgotten that hyperglycemia in tissues where glucose uptake is insulin-independent (brain, liver, kidneys, erythrocytes, leukocytes, platelets, lens of the eye, placenta) is more likely to cause metabolic disturbances [41,42]. When glucose enters the cell, it is subjected to phosphorylation; however, about 5% of glucose is not phosphorylated but is directly metabolized through the polyol pathway into sorbitol and fructose. When blood glucose levels are high, the percentage of glucose that is metabolized through the polyol pathway can increase up to 20%. In the polyol pathway, the enzyme aldose reductase (AR), with the help of the coenzyme nicotinamide adenine dinucleotide phosphate (NADPH), reduces glucose to sorbitol [21,29,43,44]. Sorbitol is then oxidized to fructose with the help of the enzyme sorbitol dehydrogenase and the coenzyme nicotinamide adenine dinucleotide (NADH) [21] (Figure 2).

Activation of the polyol pathway causes damage to blood vessels in several ways:

1. Fructose and its metabolites (trioses-phosphate, methylglyoxal, fructose-3-phosphate, and 3-deoxyglucose) lead to increased production of AGEs [29,45].
2. In the polyol pathway, the depletion of NADPH due to its conversion to NADH reduces the production of glutathione, one of the key antioxidants [29,46].
3. An increase in NADH levels causes an elevation in the levels of glycerol-3-phosphate, which activates PKC [21,24,25,29].
4. During hyperglycemia, ROS generated by AR lead to accelerated thrombus formation by activating c-Myc, inhibiting *miRNA-24*, and increasing the release of vWF from endothelial cells [21,47].

## 4. Insulin Resistance

In addition to its effects on the coagulation process and its acute and prolonged effects on the endothelial cells of blood vessels, hyperglycemia, through several different pathophysiological mechanisms, can also lead to insulin resistance.

Insulin resistance (IR) is a condition that significantly contributes to the development of macrovascular complications [41]. In the state IR state, there is reduced sensitivity of certain tissues to the physiological effects of insulin, including adipose tissue, the liver, and muscles, which leads to disturbances in glucose and lipid metabolism [41,43]. One of the roles of insulin is to stimulate lipogenesis in the liver by converting free fatty acids and glucose into triglycerides, which are enriched with very low-density lipoproteins (VLDLs) that have a high atherogenic potential [31,41,48]. However, in patients with insulin resistance, the liver continues to produce an excessive amount of VLDL, which worsens the already elevated levels of triglycerides in the blood. Insulin also stimulates the synthesis of apolipoprotein A, a key protein in high-density lipoproteins (HDLs). In a state of IR, this process is impaired, leading to a decrease in HDL-C levels [43]. Additionally, IR increases the oxidation of fatty acids, leading to increased oxidative stress in the diabetic microvasculature [31,49]. It is believed that hyperinsulinemia leads to hypertrophy and hyperplasia of the VSMC, the increased synthesis of extracellular matrix proteins, and an excessive release of free fatty acids (FFAs), which subsequently increase oxidative stress and activate PKC [31,50].

Recent studies have shown that damage to caveolin-3 (Cav-3) protein, which is found in cardiac muscle, plays a significant role in the development of insulin resistance. This protein is crucial for insulin signaling, glucose metabolism, and lipid homeostasis. Reduced expression of Cav-3 is associated with decreased glucose uptake and increased insulin resistance, which is a key pathological mechanism in the development of diabetes. Additionally, impaired expression of Cav-3 is closely associated with increased oxidative stress and heightened sensitivity to ischemia-reperfusion injury in diabetic myocardium [51].

In conditions of IR, there is reduced expression of endothelial eNOS and increased synthesis of asymmetric dimethylarginine (ADMA), which is both an endogenous and competitive inhibitor of eNOS. This reduces the vasodilatory capacity of NO. The reduced concentration of NO under insulin resistance conditions also leads to impaired function of the Na+/K+ ATPase, which results in an increased amount of intracellular calcium ions, and this, in turn, leads to the development of pathological vasoconstriction [21,52].

Research has shown that insulin resistance also increases the expression of PAI-1 and several adhesion molecules, which contribute to the progression of atherosclerotic lesions. Research has also shown that insulin resistance, in combination with Metabolic Associated Steatotic Liver Disease (MASLD), contributes to increased expression of PAI-1 and several adhesion molecules, which leads to the progression of atherosclerotic lesions, proliferation of VSMC, and the subsequent development of restenosis [16,31,42,53].

In addition to all the previously mentioned factors in the development of atherosclerosis, insulin resistance has the greatest impact through lipid metabolism disturbances [41,54].

## 5. Dyslipidemia in Diabetic Patients

Atherosclerosis can be caused by many risk factors. One of the important ones is dyslipidemia. Studies have shown that patients with type 1 diabetes and patients with type 2 diabetes have a high risk of developing lipid metabolism disorders, but dyslipidemia is more common in patients with type 2 diabetes [4,55].

In diabetics, elevated levels of triglycerides, total cholesterol, and low-density lipoprotein (LDL-C) are often detected, as are low levels of HDL-C [43]. The exact mechanism of lipoprotein abnormalities in diabetes is not fully researched, but it is believed that insulin resistance, along with the increased production of small, dense LDL particles, contributes significantly to the progression of atherosclerosis [41,43]. It is believed that native LDL particles do not have an atherogenic potential, but under conditions of hyperglycemia, their physicochemical properties change, making them atherogenic [4,56]. Under conditions of hyperglycemia, there is increased glycosylation of LDL molecules and their receptors occurs, which reduces the clearance and increases the accumulation of LDL cholesterol. Glycosylation of LDL allows for its easier oxidation under conditions of increased oxidative stress [8,9]. LDL particles can be oxidized by free radicals such as superoxide, hydroxyl radicals, and hydrochloric acid present in the extracellular matrix, as well as by the enzymatic activity of phospholipases and lipoxygenases [1,57,58]. The oxidation process is accelerated by the deficiency of protective antioxidants such as tocopherol, ascorbate, urate, and serum albumin, which has been observed in patients with diabetes [1,8,9]. In the process of atherosclerosis formation, oxLDL stimulates endothelial cells to express adhesion molecules such as ICAM-1, VCAM-1, MCP-1, and E-selectin, which mediate the movement and adhesion of leukocytes to the endothelium and their migration into the intima [8,10,12]. It has also been proven that modified LDL has a lower affinity for the LDL receptor (LDLR), and therefore it is predominantly taken up through nonspecific phagocytosis by recruited monocytes [1,4,8,9,59]. The ultimate result is the formation of foam cells, in whose cytoplasm lipid droplets are accumulated [1,7,8].

In addition to glycosylation and oxidation, LDL particles can also undergo desialization, which leads to an increase in their density, a reduction in size, and the development of a negative charge, further promoting the process of atherogenesis [4,60].

It has also been shown that hypertriglyceridemia in patients with diabetes increases platelet activation through apolipoprotein E (ApoE). Besides platelet activation, VLDL particles can also impair fibrinolysis and the entire coagulation cascade [31,61].

As previously mentioned, patients with type 2 diabetes mellitus have reduced HDL particle concentrations. The bioactive lipid sphingosine-1-phosphate (S1P), which has atheroprotective properties, is bound to HDL particles. Recent studies have shown that increased levels of S1P are associated with a reduced risk of cardiovascular events, while decreased levels of S1P in patients with type 2 diabetes (T2D) lead to impaired anti-inflammatory properties of HDL, which may contribute to the development of atherosclerosis. Reduced levels of S1P in HDL are also correlated with increased plaque calcification, likely due to reduced inhibition of pro-inflammatory cytokines IL-1β and IL-6 in macrophages [62].

### New Therapeutic Approaches in the Treatment of Dyslipidemia

Recent studies have clarified the role of Proprotein Convertase Subtilisin/Kexin type 9 (PCSK9) in lipid metabolism, demonstrating its impact on low-density lipoprotein (LDL) levels, which are a key factor in the development of atherosclerosis and associated cardiovascular events. Research shows that PCSK9 inhibitors, such as alirocumab and evolocumab, significantly reduce LDL-C levels and the risk of cardiovascular diseases in individuals with diabetes. These findings support the hypothesis that targeting PCSK9 could provide dual benefits: improving glycemic control and reducing cardiovascular morbidity. Additionally, the discovery of proteins NRP1 and CD27 offers new insights into the pathophysiology of coronary heart disease (CHD) in patients with type 2 diabetes. NRP1 plays a role in various biological processes, such as angiogenesis and neuroprotection, while CD27 is associated with immune system modulation, which may contribute to chronic inflammation. These proteins may represent key targets for innovative therapies aimed at reducing the risk of CHD in individuals with diabetes [63].

## 6. Oxidative Stress

Oxidative stress occurs due to excessive production of ROS and reduced levels of antioxidants in tissues [21,64]. The most significant ROS include the superoxide anion (O^2−^), hydrogen peroxide (H_2_O_2_), and the hydroxyl radical (OH). The main sources of ROS are mitochondrial enzymes: NADPH oxidase, xanthine oxidase (XO), lipoxygenase (LOX), and inflammatory cells (neutrophils and macrophages) [21,65].

In conditions of hyperglycemia, there is an increased production of ROS, most commonly through the formation of AGEs and through the activation of NADPH oxidase [21,66]. The activation of NADPH oxidase is significantly influenced by the previously mentioned PKC [21,24,25,29]. The action of ROS can accelerate the progression of atherosclerosis in several ways by causing endothelial cell damage, the enhanced oxidation of LDL particles, chronic inflammation, the destabilization of the atherosclerotic plaque, and thrombosis [21,49,64]. In patients with diabetes, the two most significant mechanisms by which ROS contribute to the development of atherosclerosis are glucose oxidation and enhanced glycolysis [9]. When blood sugar levels are elevated, glycolysis is enhanced, which increases the production of O2- in cells.

During hyperglycemia, ROS also stimulates VSMCs to release IL-1, Interleukin 6 (IL-6), Interleukin 1 beta (IL-1β), TNFα, histamine, and bradykinin, which disrupt the junctions between endothelial cells, increase permeability, and thereby facilitate the passage of monocytes and oxLDL [21]. These mediators also inhibit the activity of eNOS, thereby reducing the production of NO, a potent vasodilator [1,4,21,34,67].

### Non-Coding RNAs and Oxidative Stress

Non-coding RNAs play an important role in the development of oxidative stress. They are currently considered potential biomarkers and disease modulators [21,68]. The group of non-coding RNAs includes short RNAs (microRNAs, miRNAs) and long non-coding RNAs (lncRNAs), including intergenic lncRNAs (lincRNAs) [21]. Long non-coding RNAs play a significant role in the development and pathogenesis of cardiovascular and metabolic diseases, including atherosclerosis, myocardial infarction, and heart failure, acting through the regulation of cellular processes such as proliferation, hypertrophy, and apoptosis [69]. MicroRNAs are particularly distinguished in the development of atherosclerosis [21,70]. To date, over 2500 microRNAs have been identified, and several of them play a role in the pathogenesis of diabetes mellitus [21,71].

Among the most significant are *miR-146a* and *miR-126*. Regarding *miR-146a*, its expression in endothelial cells is reduced in patients with diabetes, leading to the increased expression of NADPH oxidase 4 (NOX4), thereby resulting in increased ROS production and endothelial damage [21,72]. On the other hand, the expression of *miR-126* is a risk factor for the development of type 2 diabetes. Another non-coding RNA that likely plays a role in atherosclerosis is the long non-coding RNA Dnm3os (opposite strand of dynamin 3). Excessive expression of this RNA leads to the increased production of inflammatory genes and enhanced phagocytosis of LDL by macrophages. It is also elevated in the monocytes of patients with type 2 diabetes [21,73].

On the other hand, there is a large number of microRNAs that regulate endothelial function. Among the most significant is *miR-155*, which leads to reduced expression of ET-1 and angiotensin II type I receptors (AT I), suggesting its role in protecting the endothelium [1]. Also, in the process of inhibiting endothelial permeability, an important role is played by *miR-126*, which regulates the VEGF pathway, as well as *miR-181b*, which regulates the (NF-kB) pathway [1,74,75]. One should not forget *miR-92a*, which reduces endothelial inflammation and the size of atheromatous plaques through the regulation of Kruppel-like factor 2 (KLF2) [1,76].

Therapeutic modification of microRNAs in the prevention of atherosclerosis in diabetics represents an innovative strategy aimed at regulating key pathophysiological processes. Further research is needed to ensure the safe clinical application of therapy while minimizing adverse effects. The main therapeutic approaches would focus on inhibiting pro-atherogenic microRNAs, increasing the expression of cardioprotective microRNAs, and modulating their signaling pathways to prevent and slow down the atherosclerotic process [77].

## 7. Chronic Inflammation

Atherosclerosis is a chronic inflammatory disease [32]. In patients with diabetes and atherosclerosis, several substances contribute to chronic inflammation [1,32,78]. Among the most important are AGEs, cholesterol, uric acid, and excessive activation of the NLRP3 inflammasome, which are collectively referred to as Pathogen-Associated Molecular Patterns (PAMPs). In addition to PAMPs, there are also Damage-Associated Molecular Patterns (DAMPs) [29,79]. PAMPs, DAMPs, and oxLDL are largely responsible for the formation of the active NLRP3 inflammasome, which leads to the activation of numerous cytokines, among which IL-1β and IL-18 are the most significant, playing a key role in inflammation [1,29,32,80]. IL-1β disrupts the junctions between endothelial cells, increases permeability, and thereby facilitates the penetration of oxLDL, while IL-18 leads to enhanced leukocyte adhesion, LDL oxidation, and VSMC apoptosis [32,78]. In addition to cytokine release, NLRP3 also plays a role in increasing the levels of adhesion molecules ICAM-1, VCAM-1, and E-selectin in the intima of the blood vessel, which leads to the recruitment of inflammatory cells [32,81]. Studies have shown that diabetic patients with type 2 diabetes have high levels of NLRP3 in monocytes, increased inflammasome activity, and elevated levels of IL-1β and IL-18 in peripheral blood [32,78]. Pro-inflammatory cytokines TNF-α and IL-6, which are produced in increased amounts in patients with diabetes, play a key role in the development of inflammation [1,43,44,67,82]. It is believed that TNF-α induces cytotoxicity, while IL-6 increases endothelial permeability [32,43,83]. Another key proinflammatory cytokine that should not be overlooked is galectin-3, which plays a central role in mediating the inflammatory response, particularly in the context of various pathological conditions, including cardiovascular diseases and diabetes. Increased galectin-3 concentration can contribute to the activation of inflammation, oxidative stress, induction of myocardial cell apoptosis, as well as the promotion of fibrosis and other pathological processes [84].

The inflammatory process, in addition to playing a role in the formation of the atherosclerotic plaque, also contributes to its destabilization, i.e., plaque rupture. Pro-inflammatory cytokines such as Interferon Gamma (IFN-γ) inhibit collagen formation in VSMCs, while cytokines like IL-1β, TNF-α, and CD40 ligand increase the expression of MMPs in VSMCs. Thanks to these data, it has been concluded that when inflammation predominates, the plaque becomes unstable and prone to rupture [1,85,86].

Research has shown that inflammation can also be triggered by the NF-κB. In states of hyperglycemia, its activation occurs, and it further leads to increased production of vascular adhesion molecules and cytokines, which in turn activates inflammatory cells in the blood vessel wall [31,87]. Prostaglandin E2 (PGE2) also plays a role in the development of inflammation by increasing body temperature and causing pain, while its receptors (EP receptors) contribute to plaque erosion and rupture [1,88].

According to the latest research, the attenuation of pericoronary adipose tissue (PCATa) has also been associated with the development of coronary artery disease (CAD) in patients with diabetes. PCATa reflects local inflammation in the vicinity of the coronary arteries and has shown a strong association with the presence of coronary artery disease, the emergence of high-risk atherosclerotic plaques, progression of non-calcified lesions, and an increased risk of cardiovascular mortality. Studies have demonstrated that PCATa values are significantly higher in individuals with type 2 diabetes mellitus, particularly in those who have already experienced cardiovascular events [89].

### 7.1. Chronic Inflammation, Therapeutic Dilemmas

The treatment of chronic inflammation in diabetic patients presents a challenge, as inflammation plays a key role in the development of atherosclerosis, especially in type 2 diabetes. When it comes to drug use, the application of Non-Steroidal Anti-Inflammatory Drugs (NSAIDs), (TNFα) inhibitors, Glucagon-Like Peptide-1 Receptor Agonist (GLP-1) and Sodium-Glucose Cotransporter-2 Inhibitor (SGLT2) as well as corticosteroids and statins, remains under investigation [90,91,92,93]. Currently, the drugs being studied are GLP-1 agonists and SGLT2 inhibitors, which exhibit their antioxidant and anti-inflammatory properties by reducing oxidative stress, inhibiting inflammatory pathways (e.g., NF-kB), and reducing pro-inflammatory cytokines [90,91,92]. All the aforementioned drugs may contribute to reducing inflammation but can also cause potential harmful effects.

One of the main questions in treating chronic inflammation in diabetics is how aggressively the therapy should be approached. Although numerous experiments have shown that therapies reducing inflammation can improve glycemic control and reduce the risk of cardiovascular events, disagreements still exist regarding which drugs are best suited for this purpose and what long-term effects such therapy might have. It is also important to emphasize that, in addition to drug use, changes in diet and regular physical activity play a very important role in reducing inflammation [93].

### 7.2. NETosis and Chronic Inflammation

Recent studies have discovered that hyperglycemia can also trigger the process of NETosis, a type of cell death that differs from apoptosis and necrosis [29,94]. This process occurs when neutrophils release NETs into the extracellular space’s sticky networks composed of nucleic acids, proteins, and proteases that capture pathogenic cells. Bacteria trapped in the NETs are subjected to phagocytosis by neutrophils and macrophages, but they are also killed by the NETs themselves, which possess bactericidal properties. This mechanism is highly effective for bacterial elimination but can simultaneously trigger excessive inflammation by releasing intracellular components such as nucleic acids, proteases, and proteins [29]. Several components of NETs, such as histones and various proteases, possess procoagulant properties that activate platelets and promote their aggregation, directly contributing to the formation of atherosclerotic plaques [29,54]. Other NET components, like neutrophil elastase (NE) and cathepsin G (CatG), play a key role in the breakdown of tissue factor pathway inhibitors, further promoting blood coagulation and contributing to thrombus growth [29,95]. Studies have shown that NETs can also become an integral part of the structure of an atherosclerotic plaque, depositing in the lipid core or fibrous cap. Since NETs contain numerous cytokines, proteases, and other molecules that recruit neutrophils, they significantly contribute to plaque destabilization, increasing the likelihood of its rupture and subsequent thrombosis [96].

Based on these data, NETs can be a significant therapeutic target in the prevention of atherosclerotic plaque formation due to their role in inflammation, plaque destabilization, and thrombosis. Research and development of therapeutic strategies targeting NETs may contribute to reducing the progression of atherosclerosis and lowering the risk of acute cardiovascular events.

Potential therapeutic strategies include the following:

1. Inhibition of NET formation by developing therapies that target key enzymes in the NETosis process, such as NADPH oxidase and peptidyl-arginine deiminase 4 (PAD4).
2. Increasing the elimination of NETs through the use of DNases or other enzymes that degrade DNA.
3. Neutralization of the procoagulant components of NETs, such as histones and proteases.
4. An immunological approach using antibodies that bind to NET components, aiding in their elimination from circulation and reducing their impact on plaque destabilization and thrombosis.
5. Regulation of inflammation and immune responses, including control of inflammatory pathways such as NF-kB, which can reduce the recruitment of neutrophils and other inflammatory cells [96,97].

One such drug is canakinumab, an inhibitor of inflammatory cytokines, which has shown the ability to reduce inflammation markers [4,43,98].

## 8. The Significance of the Connection Between Diabetes Mellitus and Liver Fibrosis in the Development of Atherosclerosis

Recent studies have shown that liver fibrosis in patients with type 2 diabetes mellitus (T2DM) plays a significant role in the development of cardiovascular complications. The key pathophysiological mechanisms through which liver fibrosis contributes to the development of atherosclerosis include inflammation, oxidative stress, immune changes, and increased endothelial dysfunction. The degree of liver fibrosis was assessed using the FIB-4 index, which is gaining increasing importance not only due to its association with liver fibrosis but also because of its role in immune and inflammatory mechanisms that are crucial in the development of atherosclerosis and ischemic heart disease. A recent study indicates a significant association between higher FIB-4 levels and the incidence of myocardial infarction (MI) in patients with T2DM, particularly in older individuals and those with comorbidities such as high BMI, hypertension, and coronary heart disease (CHD). Due to its high sensitivity and ease of application, FIB-4 can be used as a valuable clinical marker for identifying patients at high risk of cardiovascular complications and for adjusting personalized therapeutic strategies [99].

## 9. The Interrelationship Between Metabolic and Inflammatory Pathways in the Formation of an Atherosclerotic Plaque

In the pathogenesis of atherosclerotic plaque, in the context of hyperglycemia, the first and perhaps most significant mechanism involves the non-enzymatic glycation of proteins and lipids, which contributes to the formation of AGEs [1,4,30]. AGEs bind to their RAGE receptor, leading to the activation of several pro-inflammatory pathways and inflammatory cytokines, which increase endothelial permeability, allowing for the accumulation of lipoproteins and immune cells in the arterial intima [24,33]. Additionally, the AGE-RAGE connection results in increased production of ROS, which inhibit NO synthase, leading to reduced vasodilation and increased vascular tone [4,21,31,34].

Furthermore, ROS activate inflammatory pathways, causing chronic inflammation in the vascular endothelium, which worsens vascular function. Chronic inflammation increases endothelial permeability, enabling the accumulation of lipids and immune cells, contributing to the formation of the atherosclerotic plaque [21,49,64]. This process destabilizes the plaque, increasing the risk of rupture and the occurrence of serious cardiovascular events, such as thrombosis [1,85,86].

Based on the mechanisms outlined above, it can be concluded that hyperglycemia, oxidative stress, and inflammation act synergistically, not only in the initiation and progression of the atherosclerotic plaque but also in its destabilization. This interplay contributes to an increased risk of plaque rupture and the occurrence of severe cardiovascular events with potentially fatal outcomes.

## 10. Conclusions

It has been shown that diabetes mellitus contributes to the development and accelerated progression of atherosclerosis through various pathophysiological mechanisms. Previous studies have shown that adequate glycemic control and the reduction in risk factors (smoking, obesity, stress, high blood pressure) remain the most reliable strategies in the prevention of atherosclerosis. The role of primary prevention should not be overlooked, which involves the early diagnosis of diabetes mellitus in patients who do not have cardiovascular diseases. When it comes to therapeutic measures, the use of salicylates in primary prevention in diabetic patients remains a topic of debate due to the balance between potential benefits and risks. Although some studies suggest that their anti-inflammatory and antiplatelet properties may reduce the risk of thrombosis in individuals with type 2 diabetes, there are also significant side effects, such as gastrointestinal bleeding. Additionally, certain studies indicate a limited effect of salicylates in primary prevention. In accordance with the recommendations of the AHA and WHO, their use should be selective, taking into account the individual cardiovascular disease risk. In the end, the data from the previously mentioned DCCT and EDIC studies should not be overlooked, as they emphasize the importance of early intervention in diabetes management to reduce the risk of cardiovascular damage. These studies highlight that later normalization of glycemia cannot fully undo the negative consequences of early periods of hyperglycemia.
